# Impact of HACOR Score on Noninvasive Ventilation Failure in Non-COPD Patients with Acute-on-Chronic Respiratory Failure

**DOI:** 10.1155/2021/9960667

**Published:** 2021-07-22

**Authors:** Min Ding, Xiaoli Han, Linfu Bai, Shicong Huang, Jun Duan

**Affiliations:** Department of Respiratory and Critical Care Medicine, The First Affiliated Hospital of Chongqing Medical University, Chongqing, China

## Abstract

**Background:**

A rating scale that takes into account heart rate, acidosis, consciousness, oxygenation, and respiratory rate (the HACOR score) has been used to predict noninvasive ventilation (NIV) failure in patients with chronic obstructive pulmonary disease (COPD). However, the HACOR score has not been used to predict NIV failure in non-COPD patients with acute-on-chronic respiratory failure.

**Methods:**

This study was performed in the respiratory intensive care unit of a teaching hospital. Data had been collected prospectively between June 2011 and January 2019. We enrolled non-COPD patients who received NIV due to acute-on-chronic respiratory failure, pH < 7.35, and PaCO_2_ >45 mmHg. NIV failure was defined as requiring intubation or dying during NIV. The HACOR score was determined at initiation and after 1-2, 12, and 24 h of NIV. Scores can range from 0 to 27, with higher scores indicating a higher risk of NIV failure.

**Results:**

A total of 148 patients were enrolled in the study, 52 with sleep apnea-hypopnea syndrome, 34 with chronic thoracic sequelae, 31 with bronchiectasis, 14 with chest wall deformity, 5 with obesity-hypoventilation syndrome, and 12 with other conditions. Of the patients, 19 (13%) experienced NIV failure. From initiation to 24 h of NIV, the HACOR scores of patients who experienced NIV failure were much higher than those of patients who received successful NIV. The area under the receiver operating characteristic curve was 0.69, 0.91, 0.91, and 0.94 when the HACOR score was tested at initiation and after 1-2, 12, and 24 h of NIV, respectively. To obtain the best sensitivity and specificity, the cutoff value at initiation was 7 with a sensitivity of 68% and a specificity of 61%. After 1-2 h of NIV, it was 5 with a sensitivity of 90% and a specificity of 85%. After 12 h of NIV, it was 4 with a sensitivity of 82% and a specificity of 91%. After 24 h of NIV, it was 2 with a sensitivity of 100% and a specificity of 76%.

**Conclusions:**

The HACOR score has high sensitivity and specificity for predicting NIV failure among non-COPD patients who receive NIV due to acute-on-chronic respiratory failure with respiratory acidosis.

## 1. Introduction

Physiologic research shows that noninvasive ventilation (NIV) increases minute ventilation, improves gas exchange, counterbalances intrinsic positive end-expiratory pressure (PEEP), and decreases the work of breathing [1, 2]. In patients with hypoxemic or hypercapnic respiratory failure, NIV reduces the requirement for intubation for invasive mechanical ventilation [2–5]. As NIV benefits patients with acute respiratory failure, its use increases year by year [6].

Acute-on-chronic respiratory failure is common in patients with chronic obstructive pulmonary disease (COPD), sleep apnea-hypopnea syndrome, chronic thoracic sequelae, bronchiectasis, chest wall deformity, obesity-hypoventilation syndrome, neuromuscular disease, and other conditions. In COPD patients with hypercapnia due to acute-on-chronic respiratory failure, NIV reduces the need for intubation [7, 8]. Guidelines strongly recommend providing NIV to COPD patients [9, 10]. However, evidence of the use of NIV is rare among non-COPD patients with acute-on-chronic respiratory failure.

Although NIV reduces the need for intubation among COPD patients, mortality increases significantly if patients experience NIV failure [11, 12]. Among patients who experience NIV failure, delayed intubation further increases mortality [13]. Therefore, early identification of patients at risk for NIV failure and early intubation may reduce mortality. In a previous study, we developed a rating scale (the HACOR score) to predict the risk of NIV failure in COPD patients who experienced acute-on-chronic respiratory failure [14]. It takes into account heart rate, acidosis (assessed by pH), consciousness (assessed by Glasgow Coma Scale (GCS) score), oxygenation, and respiratory rate. The HACOR score has high sensitivity and specificity for predicting NIV failure in COPD patients. As the pathophysiologic mechanism of acute-on-chronic respiratory failure is similar in COPD and non-COPD patients, we hypothesized that the HACOR score would also have high sensitivity and specificity for predicting NIV failure among non-COPD patients with acute-on-chronic respiratory failure.

## 2. Methods

This study was performed in the respiratory intensive care unit (ICU) of a teaching hospital. Data had been collected prospectively between June 2011 and January 2019. The study protocol was approved by the local ethics committee of the First Affiliated Hospital of Chongqing Medical University. As the study was observational nature, informed consent was waived. Patients who received NIV due to hypercapnic respiratory failure were candidates for inclusion in the study. The inclusion criteria were (1) acute-on-chronic respiratory failure with respiratory acidosis, (2) use of NIV as a first-line therapy, (3) PaCO_2_ >45 mmHg, and (4) pH < 7.35. The exclusion criteria were (1) respiratory failure caused by exacerbation of COPD, (2) prophylactic use of NIV after extubation, (3) rescue use of NIV due to respiratory failure after extubation, (4) accidental extubation and use of NIV, and (5) use of a high-flow nasal cannula before or after NIV.

NIV (BiPAP Vision or V60; Philips Respironics, Carlsbad, CA, USA) was managed by attending physicians, respiratory therapists, and nurses in charge. The ventilator settings were based on the previously published protocols [14, 15]. Bilevel positive airway pressure (S/T mode) was used for all patients. PEEP was initially set at 4 cmH_2_O and titrated to counterbalance the intrinsic PEEP. Support pressure was set at 8 cmH_2_O and was increased by 2 cmH_2_O at a time to obtain a tidal volume >6 mL/kg or to reach the maximum level tolerated by the patient. The fraction of inspired oxygen was titrated to maintain SpO_2_ around 95%. The ventilator settings were adjusted based on PaCO_2_ and the severity of the patient's distress.

If respiratory failure abated, weaning from NIV was considered. NIV was used intermittently until the patient could breathe normally without ventilation. However, if respiratory failure worsened and escalation therapy was required, intubation was performed. The criteria for intubation were persistent respiratory distress with a respiratory rate >35 breaths/min, failure to correct respiratory acidosis, an inability to maintain PaO_2_/FiO_2_ above 100 mmHg, the development of conditions necessitating intubation to protect the airway (coma or seizure disorders) or to manage copious tracheal secretions, hemodynamic instability without response to fluids and vasoactive agents, and respiratory or cardiac arrest [14]. If a patient met the criteria for intubation but the attending physician did not think they would benefit from it, NIV was continued. NIV failure was defined as requiring intubation or dying during NIV [14].

Demographic data, including data on age, sex, Acute Physiology and Chronic Health Evaluation II (APACHE II) score, diagnosis, and underlying disease, were collected before the use of NIV. Data on respiratory rate, heart rate, systolic blood pressure, diastolic blood pressure, consciousness, and arterial blood gas were collected at initiation and after 1-2, 12, and 24 h of NIV. Data on the support pressure and PEEP of the ventilator were collected after 1-2, 12, and 24 h of NIV. Patients were followed up to discharge or death, whichever came first. Data on the duration of NIV, the length of stay in the ICU, and the length of stay in the hospital were also collected.

The HACOR score was determined before and after 1-2, 12, and 24 h of NIV [14]. Heart rate <100, 100–119, 120–139, and >139 beats per minute was given 0, 1, 2, and 3 points, respectively. Acidosis was assessed by pH. pH ≥ 7.35, 7.30–7.34, 7.25–7.29, 7.20–7.24, and <7.20 was given 0, 2, 3, 5, and 8 points, respectively. Consciousness was assessed with the GCS score. GCS score of 15, 14, 13, 12, and <12 was given 0, 2, 4, 6, and 11 points, respectively. Oxygenation was assessed with PaO_2_/FiO_2_. PaO_2_/FiO_2_ ≥150, 101–149, and ≤100 was given 0, 1, and 2 points, respectively. Respiratory rate <30, 31–34, 35–39, and ≥40 breaths per minute was given 0, 1, 2, and 3 points, respectively. The HACOR score was the sum of the points for the five variables. Scores can range from 0 to 27, with higher scores indicating a higher risk of NIV failure.

In our study, 3 out of 148 patients (2%) had missing data for at least one variable. Multiple imputations were performed. The imputed value was the average of five imputations. Continuous variables were expressed as means and standard deviations or medians and interquartile ranges when appropriate. Differences between groups were tested with independent samples *t* tests or Mann–Whitney *U* tests. Qualitative variables were expressed as numbers of events (%), and differences between groups were tested with chi-square or Fisher exact probability tests. The ability to predict NIV failure was tested with the area under the receiver operating characteristic curve (AUC). The optimal cutoff value was determined when the maximal Youden index was reached [16]. We ran 1000 bootstrap samples to estimate the odds ratio (OR) and 95% confidence interval (CI) of NIV failure per 1-point increment for internal validation. A two-sided *p* < 0.05 was considered significant.

## 3. Results

**Figure 1:**
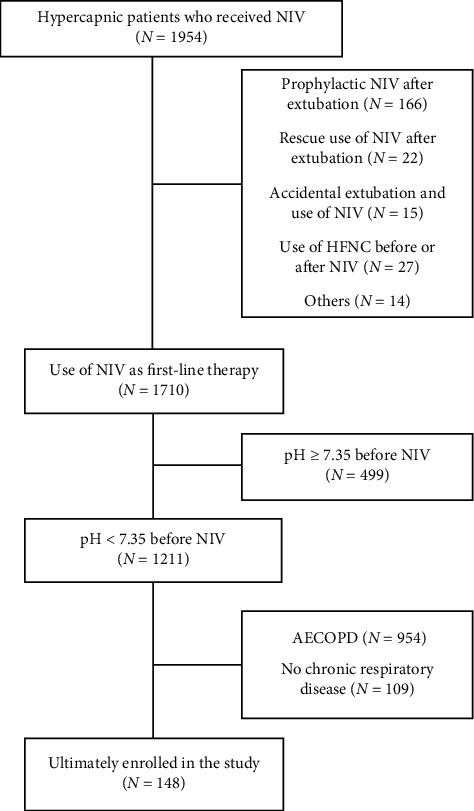
Flowchart of patient enrollment. NIV, noninvasive ventilation; HFNC, high-flow nasal cannula; AECOPD, acute exacerbation of COPD.

A total of 1954 NIV patients with hypercapnic respiratory failure were screened (Figure 1). Ultimately, 148 non-COPD patients with acute-on-chronic respiratory failure were enrolled, 52 with sleep apnea-hypopnea syndrome, 34 with chronic thoracic sequelae, 31 with bronchiectasis, 14 with chest wall deformity, 5 with obesity-hypoventilation syndrome, and 12 with other conditions. Of the 148 cases, 19 (13%) experienced NIV failure, including 2 who died during NIV (Table 1). Among the overall cohort, the median duration of NIV was 96 h, and hospital mortality was 10%.

There were no differences in age, sex, diagnosis, underlying disease, or prevalence of chronic respiratory conditions between patients who experienced successful NIV and NIV failure (Table 1). Support pressure and PEEP were also not different when recorded after 1-2, 12, and 24 h of NIV (Table 2). Before NIV, however, patients who later experienced NIV failure had a higher APACHE II score (20 ± 7 vs. 16 ± 5), a higher heart rate (122 ± 23 vs. 107 ± 22 bpm), and a lower pH (7.22 ± 0.07 vs. 7.26 ± 0.07) than those who experienced successful NIV. They also had a higher heart rate, a lower GCS score, a lower pH, and a lower PaO_2_/FiO_2_ after 1-2, 12, and 24 h of NIV.

**Figure 2:**
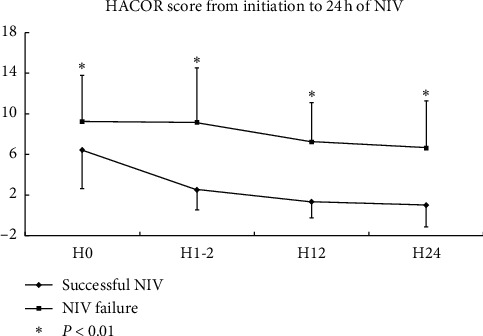
Differences in the HACOR score from initiation to 24 h of NIV between patients who experienced successful NIV and NIV failure. HACOR, heart rate, acidosis, consciousness, oxygenation, and respiratory rate; NIV, noninvasive ventilation; H0, initiation; H1-2, 1-2 h of NIV; H12, 12 h of NIV; H24, 24 h of NIV.

**Figure 3:**
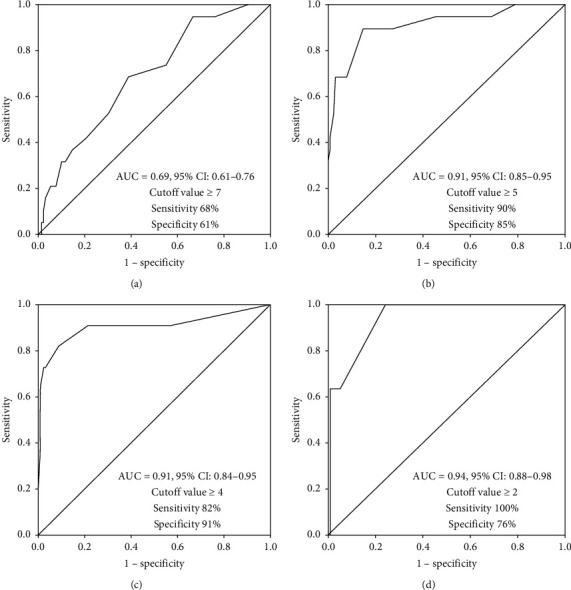
The predictive power of NIV failure assessed by the HACOR score from initiation to 24 h of NIV. HACOR, heart rate, acidosis, consciousness, oxygenation, and respiratory rate; NIV, noninvasive ventilation; AUC, area under the receiver operating characteristic curve; CI, confidence interval.

The HACOR score was much lower in patients who experienced successful NIV than in patients with NIV failure when it was measured at initiation and after 1-2, 12, and 24 h of NIV (Figure 2). The OR for NIV failure was 1.15, 1.99, 2.14, and 1.53 per 1-point increment when the HACOR score was assessed at initiation and after 1-2, 12, and 24 h of NIV, respectively (Table 3). To predict NIV failure, the AUC was 0.69, 0.91, 0.91, and 0.94 when the HACOR score was assessed at initiation and after 1-2, 12, and 24 h of NIV, respectively (Figure 3). To obtain the best sensitivity and specificity, the cutoff value at initiation was 7 with a sensitivity of 68% and a specificity of 61%. After 1-2 h of NIV, it was 5 with a sensitivity of 90% and a specificity of 85%. After 12 h of NIV, it was 4 with a sensitivity of 82% and a specificity of 91%. After 24 h of NIV, it was 2 with a sensitivity of 100% and a specificity of 76%.

## 4. Discussion

The rate of NIV failure was low in this sample of non-COPD patients with acute-on-chronic respiratory failure with respiratory acidosis. The HACOR score, which takes into account heart rate, acidosis, consciousness, oxygenation, and respiratory rate, had high sensitivity and specificity for predicting NIV failure when it was measured within 24 h of NIV. A higher HACOR score was associated with an increased risk of NIV failure.

NIV is widely used in patients with acute-on-chronic respiratory failure. The spectrum of disease includes COPD, sleep apnea-hypopnea syndrome, chronic thoracic sequelae, bronchiectasis, chest wall deformity, obesity-hypoventilation syndrome, neuromuscular disease, and other conditions [17]. The use of NIV is strongly recommended for patients with COPD regardless of patients' stability or acute-on-chronic respiratory failure [9, 10, 17]. However, the effect of NIV in non-COPD populations is lacking. To the best of our knowledge, this is the largest study to describe the characteristics of non-COPD patients who received NIV due to acute-on-chronic respiratory failure with respiratory acidosis. Our study showed a rate of NIV failure of only 13%, which indicates that NIV can be used successfully with the majority of non-COPD patients who experience acute-on-chronic respiratory failure with respiratory acidosis.

The HACOR score was developed by our team with COPD patients who received NIV due to acute-on-chronic respiratory failure [14]. It takes into account heart rate, acidosis, consciousness, oxygenation, and respiratory rate. It has excellent power to predict NIV failure in COPD patients. However, its accuracy at predicting NIV failure in non-COPD patients with acute-on-chronic respiratory failure with respiratory acidosis is not known. The current study validated the use of the HACOR score with these patients and found very good sensitivity and specificity for predicting NIV failure. Therefore, the HACOR score can be used to predict NIV failure in both COPD and non-COPD patients who experience acute-on-chronic respiratory failure with respiratory acidosis.

NIV failure significantly increases the risk of death [11, 12]. Our study confirms this. We found that mortality was 53% in patients who experienced NIV failure but only 3% in patients who had successful NIV. Reducing mortality is challenging. Our previous study showed that patients with a high risk of NIV failure identified by the HACOR score who were intubated early had lower mortality than those whose intubation was delayed [14]. Therefore, early identification of the risk of NIV failure and early intubation in non-COPD patients with acute-on-chronic respiratory failure may help reduce mortality. The current study shows that the HACOR score is a simple and reproducible tool for predicting NIV failure. The optimal cutoff values to obtain the best sensitivity and specificity were 7, 5, 4, and 2 at initiation and after 1-2, 12, and 24 h of NIV, respectively. The HACOR score is a good tool for clinical staff to use to manage non-COPD patients who require NIV due to acute-on-chronic respiratory failure.

Our study has several limitations. First, we screened hypercapnic patients admitted to our ICU within the past 8 years, and only 148 non-COPD patients with acute-on-chronic respiratory failure were enrolled. The characteristics of the non-COPD patients in the study varied greatly. It was impossible to describe the characteristics of each subgroup given the small sample sizes. A larger sample is needed to perform subgroup analyses. Second, this was an observational study performed in a single center. The results must be validated for other centers. Third, COPD is frequently underdiagnosed in the real word [18]. We were unable to exclude all cases of COPD from our study because of a lack of data on patients' smoking history, previous hospitalizations due to respiratory failure, and pulmonary function. Further study with stricter assessment is required to exclude cases of underdiagnosed COPD.

## 5. Conclusions

The rate of NIV failure is low in non-COPD patients who experience acute-on-chronic respiratory failure with respiratory acidosis. Among these patients, the HACOR score has high sensitivity and specificity for predicting NIV failure.

## Figures and Tables

**Table 1:** Baseline data for patients who experienced successful NIV and NIV failure.

	Overall cohort, *N* = 148	Successful NIV, *N* = 129	NIV failure, *N* = 19	*P*
Age, years	64 ± 16	64 ± 16	67 ± 11	0.49
Sex, male	83 (56%)	71 (55%)	12 (63%)	0.62
APACHE II score	16 ± 5	15 ± 4	20 ± 7	<0.01

Diagnosis				
Sleep apnea-hypopnea syndrome	52 (35%)	48 (37%)	4 (21%)	0.12
Chronic thoracic sequelae	34 (23%)	28 (22%)	6 (32%)	
Bronchiectasis	31 (21%)	25 (19%)	6 (32%)	
Chest wall deformity	14 (10%)	14 (11%)	0 (0%)	
Obesity-hypoventilation syndrome	5 (3%)	3 (2%)	2 (11%)	
Others	12 (8%)	11 (9%)	1 (5%)	

Underlying disease				
Hypertension	68 (46%)	62 (48%)	6 (32%)	0.22
Chronic heart disease	29 (20%)	25 (19%)	4 (21%)	>0.99
Diabetes mellitus	28 (19%)	23 (18%)	5 (26%)	0.36
Chronic renal failure	11 (7%)	9 (7%)	2 (11%)	0.63
Liver cirrhosis	4 (3%)	2 (2%)	2 (11%)	0.08
Duration of NIV (h)	96 (42–143)	103 (50–166)	29 (3–77)	<0.01
Length of stay in the ICU (days)	5.8 (3.7–10.8)	5.5 (3.7–10.4)	6.6 (3.4–13.2)	0.79
Length of stay in the hospital (days)	11.8 (6.8–19.0)	11.8 (6.8–18.9)	12.1 (7.6–21.8)	0.88
Hospital mortality	14 (10%)	4 (3%)	10 (53%)	<0.01

NIV, noninvasive ventilation; APACHE II, Acute Physiology and Chronic Health Evaluation II; ICU, intensive care unit.

**Table 2:** Vital signs and ventilator parameters from initiation to 24 h of NIV for patients who experienced successful NIV and NIV failure.

	Successful NIV	NIV failure	*P*
Before NIV			
Heart rate (bpm)	107 ± 22	122 ± 23	<0.01
Respiratory rate (bpm)	29 ± 6	28 ± 5	0.68
Mean arterial blood pressure (mmHg)	101 ± 16	103 ± 22	0.76
GCS score	14.5 ± 1.2	14.2 ± 1.2	0.38
pH	7.26 ± 0.07	7.22 ± 0.07	0.01
PaCO_2_ (mmHg)	81 ± 18	77 ± 17	0.28
PaO_2_/FiO_2_, (mmHg)	199 ± 99	173 ± 79	0.28

1-2 h of NIV			
Heart rate (bpm)	96 ± 18	111 ± 26	<0.01
Respiratory rate (bpm)	23 ± 5	25 ± 6	0.07
Mean arterial blood pressure (mmHg)	91 ± 13	103 ± 19	<0.01
GCS score	14.7 ± 0.7	13.7 ± 1.3	<0.01
pH	7.35 ± 0.05	7.26 ± 0.10	<0.01
PaCO_2_ (mmHg)	68 ± 18	74 ± 22	0.25
PaO_2_/FiO_2_ (mmHg)	223 ± 63	169 ± 70	<0.01
Support pressure (cmH_2_O)	17 ± 4	17 ± 4	0.58
PEEP (cmH_2_O)	6 ± 2	6 ± 2	0.47

12 h of NIV			
Heart rate (bpm)	89 ± 16	113 ± 31	<0.01
Respiratory rate (bpm)	22 ± 4	22 ± 3	0.85
Mean arterial blood pressure (mmHg)	88 ± 11	91 ± 14	0.48
GCS score	14.8 ± 0.5	14.5 ± 0.7	0.02
pH	7.38 ± 0.05	7.27 ± 0.12	<0.01
PaCO_2_ (mmHg)	65 ± 15	71 ± 22	0.19
PaO_2_/FiO_2_ (mmHg)	241 ± 86	182 ± 64	0.03
Support pressure (cmH_2_O)	18 ± 4	18 ± 3	0.89
PEEP (cmH_2_O)	7 ± 3	6 ± 2	0.53

24 h of NIV			
Heart rate (bpm)	87 ± 17	105 ± 30	<0.01
Respiratory rate (bpm)	23 ± 4	25 ± 6	0.11
Mean arterial blood pressure (mmHg)	90 ± 12	92 ± 23	0.61
GCS score	14.9 ± 0.9	14.2 ± 0.8	0.02
pH	7.40 ± 0.07	7.29 ± 0.14	<0.01
PaCO_2_ (mmHg)	59 ± 15	73 ± 33	0.01
PaO_2_/FiO_2_ (mmHg)	256 ± 80	171 ± 68	<0.01
Support pressure (cmH_2_O)	19 ± 4	20 ± 3	0.78
PEEP (cmH_2_O)	7 ± 3	6 ± 2	0.32

NIV, noninvasive ventilation; GCS, Glasgow Coma Scale; PEEP, positive end-expiratory pressure. Differences between the two groups were analyzed with independent samples *t* tests.

**Table 3:** Odds ratios for NIV failure tested by the HACOR score (per 1-point increment).

	OR (95% CI)	OR (95% CI) under 1000 bootstraps
Before NIV	1.15 (1.04–1.28)	1.15 (1.04–1.31)
1-2 h of NIV	1.99 (1.50–2.64)	1.99 (1.59–3.28)
12 h of NIV	2.14 (1.52–3.02)	2.14 (1.60–6.19)
24 h of NIV	1.53 (1.18–1.98)	1.53 (1.15–3.85)

HACOR, heart rate, acidosis, consciousness, oxygenation, and respiratory rate; OR, odds ratio; CI, confidence interval; NIV, noninvasive ventilation.

## Data Availability

The datasets analyzed during this study are available from the corresponding author upon request.

## Abbreviations

COPD: Chronic obstructive pulmonary disease
AECOPD: Acute exacerbation of COPD
NIV: Noninvasive ventilation
GCS: Glasgow Coma Scale
OR: Odds ratio
CI: Confidence interval
ICU: Intensive care unit
HACOR: Heart rate, acidosis, consciousness, oxygenation, and respiratory rate
HFNC: High-flow nasal cannula
AUC: Area under the receiver operating characteristic curve.
